# A Case of Infiltrative Hepatocellular Carcinoma With Vascular Invasion Into the Heart

**DOI:** 10.7759/cureus.17859

**Published:** 2021-09-09

**Authors:** Chimaobi M Anugwom, Johnstone Kayandabila, Siobhan Flanagan, Jose D Debes

**Affiliations:** 1 Gastroenterology, Health Partners Digestive Care, St. Paul, USA; 2 Division of Gastroenterology, Hepatology and Nutrition, University of Minnesota, Minneapolis, USA; 3 Gastroenterology, Arusha Lutheran Medical Center, Arusha, TZA; 4 Radiology, University of Minnesota, Minneapolis, USA; 5 Gastroenterology and Hepatology, University of Minnesota, Minneapolis, USA

**Keywords:** hepatocellular carcinoma, invasion, hepatitis b, sub-saharan arica, liver

## Abstract

Hepatocellular carcinoma (HCC) represents a major cause of morbidity and mortality in the world, but this problem is especially significant in low-resource countries where HCC screening recommendations and strategies are limited. We describe a rare complication of newly diagnosed HCC in a patient with untreated hepatitis B virus (HBV) infection, underscoring the importance of improving screening strategies and promoting early diagnosis of HCC in Sub-Saharan Africa.

## Introduction

Hepatocellular carcinoma (HCC) is a substantial cause of morbidity and mortality as it is the most common primary liver malignancy and the third leading cause of cancer-related death worldwide [1]. Its distribution varies geographically and is dependent on the etiology of underlying liver disease [2]. In Africa, there is a preponderance of late diagnosis of HCC and therefore, advanced disease [3]. This leads to limitations in the treatment modalities available to treat these patients. We present a case of a patient, previously diagnosed with hepatitis B virus (HBV) infection, who presented with infiltrative HCC complicated by vascular invasion to the heart.

## Case presentation

**Figure 1:**
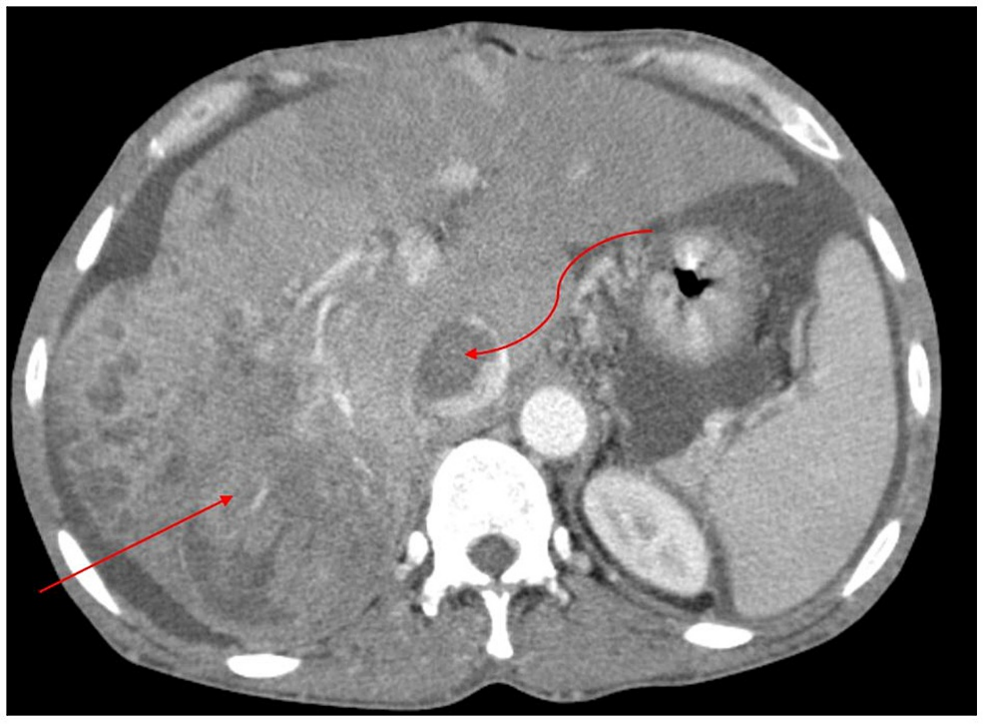
Axial early portal venous phase post-contrast CT image through the liver shows a heterogeneous mass in the right hepatic lobe, with areas of contrast washout (thin, straight arrow) consistent with hepatocellular carcinoma. The mass is seen invading the hepatic venous drainage into the inferior vena cava (curved arrow).

**Figure 2:**
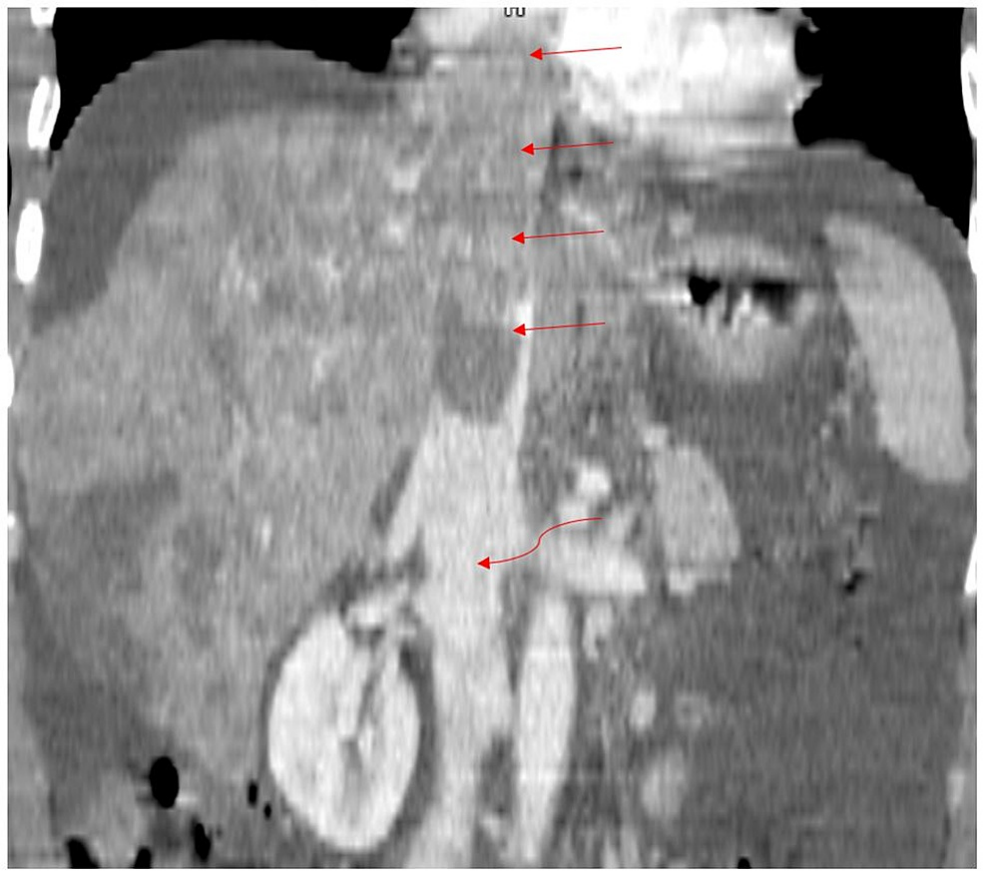
Coronal reformatted post-contrast CT demonstrating extensive tumor with invasion into hepatic venous drainage.

**Figure 3:**
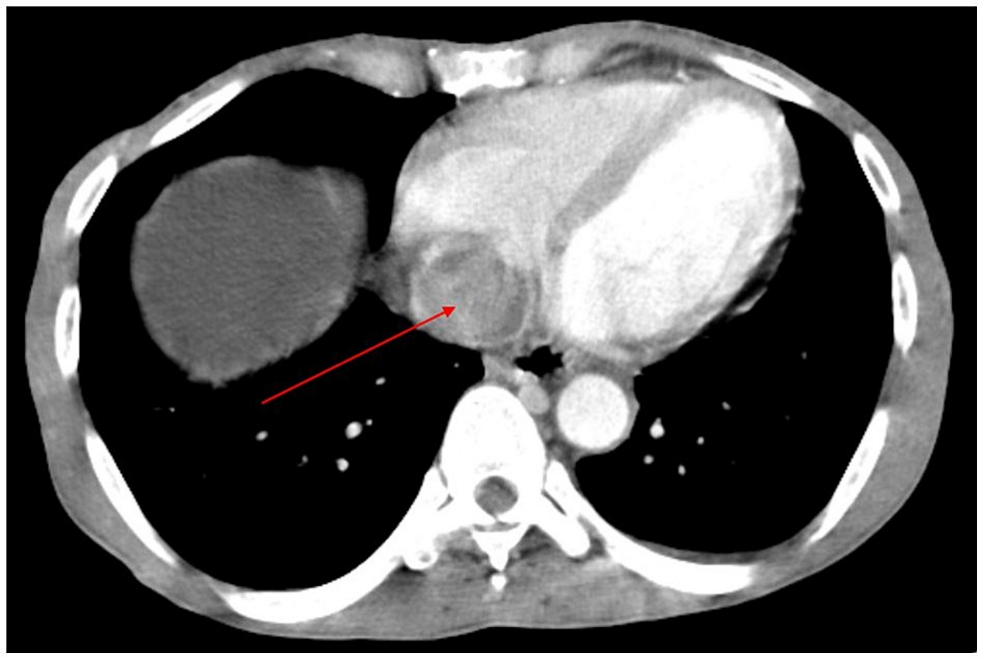
Axial post-contrast CT image through the inferior chest demonstrates tumor in the right atrium (straight arrow).

A 59-year-old male who was previously diagnosed with untreated HBV infection, with no follow up and no diagnosis of cirrhosis, presented to the emergency room with a one-week history of abdominal pain. He was lethargic and weak, but denied fever, nausea or vomiting. Physical examination noted normal blood pressure and slight tachycardia. He had right-sided abdominal fullness and mild tenderness in the right upper quadrant of his abdomen, but had no abnormal chest examination findings and no asterixis. Routine laboratory evaluation showed normal serum liver transaminases and bilirubin, but his alkaline phosphatase (ALP) was elevated to 340 IU/L. He had normal platelet count and normal international normalized ratio. Given the abdominal pain, an abdominal computed tomography (CT) scan with intravenous contrast was obtained, revealing a heterogeneous infiltrating mass occupying most of the liver, with extensive tumor invasion into the inferior vena cava and right atrium (Figures 1-3).

Based on the arterial enhancement and venous washout of the tumor, it was defined as Liver Imaging Reporting and Data System (LI-RADS) 5 consistent with HCC with locally advancing and infiltrative disease. The patient was further referred to Oncology in an outside institution for follow up and discussion of prognosis. However, the patient was again lost to follow up.

## Discussion

This is a case of advanced HCC, presenting first time with vascular invasion and tumor extension into the right atrium. Although significantly advanced disease is not rare, the late diagnosis and advanced presentation in this patient underscores the immense healthcare burden of HCC and most other chronic illnesses in Africa [1]. HCC remains a significant cause of morbidity and mortality worldwide [2,3]. In Africa, the public health burden of HCC is even greater due to the relative unawareness of underlying liver diseases such as HBV, as well as the lack of resources for prevention and management of risk factors [4,5]. Viral hepatitis remains the most common risk factor for the development of HCC worldwide, with HBV being more common in most parts of Africa [6,7].

Our patient represents a late diagnosis of HCC which typically limits the treatment options to palliative therapy and end-of-life comfort cares. Commencement of HBV therapy in the appropriate individuals reduces the risk of HCC but does not eliminate this risk completely [8]. Early and prompt screening is vital to ensure early diagnosis of HCC and potential for curative treatment [8,9]. Furthermore, invasive HCC diagnosed in his 50s suggests an early onset of disease. This, though perturbing, is not uncommon in the region as recent studies from our group in Sub-Saharan Africa have documented development of HCC in patients less than 40 years of age in almost 40% of reported cases [4,10]. This is likely contributed by the presence of aflatoxin exposure, which in individuals with HBV infection can lead to mutation of the TP53 genes and subsequent early tumor development [4].

## Conclusions

We recognize that late diagnosis of HCC, such as was seen in our patient, is relatively common in Africa and largely responsible for the morbidity and mortality of the disease. Provision of adequate resources, immunization programs, and adequate screening of at-risk individuals will be pivotal in reducing the burden of HCC in Africa.

## References

[1] Singal AG, Lampertico P, Nahon P (2020). Epidemiology and surveillance for hepatocellular carcinoma: new trends. J Hepatol.

[2] Jinjuvadia R, Salami A, Lenhart A, Jinjuvadia K, Liangpunsakul S, Salgia R (2017). Hepatocellular carcinoma: a decade of hospitalizations and financial burden in the United States. Am J Med Sci.

[3] Ferlay J, Shin HR, Bray F, Forman D, Mathers C, Parkin DM (2010). Estimates of worldwide burden of cancer in 2008: GLOBOCAN 2008. Int J Cancer.

[4] Ladep NG, Lesi OA, Mark P (2014). Problem of hepatocellular carcinoma in West Africa. World J Hepatol.

[5] Debes JD, Kayandabila J, Pogemiller H (2016). Knowledge of hepatitis B transmission risks among health workers in Tanzania. Am J Trop Med Hyg.

[6] Yang JD, Mohamed EA, Aziz AO (2017). Characteristics, management, and outcomes of patients with hepatocellular carcinoma in Africa: a multicountry observational study from the Africa Liver Cancer Consortium. Lancet Gastroenterol Hepatol.

[7] Mittal S, El-Serag HB (2013). Epidemiology of hepatocellular carcinoma: consider the population. J Clin Gastroenterol.

[8] Anugwom CM, Allaire M, Akbar SM, Sultan A, Bollipo S, Mattos AZ, Debes JD (2021). Hepatitis B-related hepatocellular carcinoma: surveillance strategy directed by immune-epidemiology. Hepatoma Res.

[9] Marrero JA, Kulik LM, Sirlin CB (2018). Diagnosis, staging, and management of hepatocellular carcinoma: 2018 practice guidance by the American Association for the Study of Liver Diseases. Hepatology.

[10] Sultan A, Anugwom CM, Wondifraw Z, Braimoh GA, Bane A, Debes JD (2020). Single center analysis of therapy and outcomes of hepatocellular carcinoma in Sub-Saharan Africa. Expert Rev Gastroenterol Hepatol.

